# Endocardial-to-mesenchymal transformation and mesenchymal cell colonization at the onset of human cardiac valve development

**DOI:** 10.1242/dev.133843

**Published:** 2016-02-01

**Authors:** Michael G. Monaghan, Miriam Linneweh, Simone Liebscher, Ben Van Handel, Shannon L. Layland, Katja Schenke-Layland

**Affiliations:** 1 Department of Women's Health, Research Institute for Women's Health, Eberhard Karls University Tübingen, 72076 Tübingen, Germany; 2 Department of Cell and Tissue Engineering, Fraunhofer Institute for Interfacial Engineering and Biotechnology (IGB), 70569 Stuttgart, Germany; 3 Department of Medicine/Cardiology, Cardiovascular Research Laboratories (CVRL), University of California Los Angeles (UCLA), Los Angeles, CA 90095, USA

**Keywords:** NFATc-1, EndMT, Heart, Semilunar valves, Extracellular matrix, Periostin

## Abstract

The elucidation of mechanisms in semilunar valve development might enable the development of new therapies for congenital heart disorders. Here, we found differences in proliferation-associated genes and genes repressed by VEGF between human semilunar valve leaflets from first and second trimester hearts. The proliferation of valve interstitial cells and ventricular valve endothelial cells (VECs) and cellular density declined from the first to the second trimester. Cytoplasmic expression of NFATC1 was detected in VECs (4 weeks) and, later, cells in the leaflet/annulus junction mesenchyme expressing inactive NFATC1 (5.5-9 weeks) were detected, indicative of endocardial-to-mesenchymal transformation (EndMT) in valvulogenesis. At this leaflet/annulus junction, CD44^+^ cells clustered during elongation (11 weeks), extending toward the tip along the fibrosal layer in second trimester leaflets. Differing patterns of maturation in the fibrosa and ventricularis were detected via increased fibrosal periostin content, which tracked the presence of the CD44^+^ cells in the second trimester. We revealed that spatiotemporal NFATC1 expression actively regulates EndMT during human valvulogenesis, as early as 4 weeks. Additionally, CD44^+^ cells play a role in leaflet maturation toward the trilaminar structure, possibly via migration of VECs undergoing EndMT, which subsequently ascend from the leaflet/annulus junction.

## Abstract (precis)

**Summary:** Global gene expression analyses on semilunar valve leaflets from embryonic human hearts reveals significant differences in the expression profiles of EndMT-associated and proliferation-associated genes.

## INTRODUCTION

Congenital heart disorders, which include aortic and pulmonary valve disease, are one of the most prevalent birth defects in humans (Roger et al., 2012). Valve malformation can lead to stenosis or calcification, which can further develop into more debilitating diseases including congestive heart failure (Fedak et al., 2002). The clinical importance of understanding valve development, valvulogenesis, is understood, yet the mechanisms underlying normal human fetal valvulogenesis are not fully elucidated (Lin et al., 2012). To date, most developmental studies define mechanisms of valvulogenesis in zebrafish, mouse or chicken models (Butcher and Markwald, 2007; de Vlaming et al., 2012; Lin et al., 2012). Such investigations have identified key mechanisms and regulatory pathways; however, these data have yet to be corroborated in humans.

Semilunar valvulogenesis begins within the first 4 weeks of development and originates from the endocardial cushions. Initially, these cushions derive from the cardiac jelly that is formed between the myocardial and endocardial layers within the distal aspect of the outflow tract (OFT) (Eisenberg and Markwald, 1995; Markwald et al., 1977; Srivastava, 2006). Cells contributing to semilunar valve formation originate from the second heart field (SHF) and the neural crest (Srivastava, 2006). During endocardial cushion formation, endocardial cells transition to a mesenchymal phenotype by a process called endocardial-to-mesenchymal transformation (EndMT) and migrate into the cardiac jelly (Armstrong and Bischoff, 2004). Afterwards, the thick endocardial cushions, which act as primitive valves, elongate into thin fibrous leaflets that exhibit a typical trilaminar extracellular matrix (ECM) structure (Armstrong and Bischoff, 2004; Combs and Yutzey, 2009; Lincoln et al., 2004; Markwald et al., 1977).

Little has been clarified regarding the mechanisms that lead to the elongation of the human leaflet. It is known from model systems that the spatial and temporal balance of cell proliferation and apoptosis in mesenchymal, endocardial and SHF-derived myocardial cells is pivotal for normal remodeling, elongation and maturation processes (Rentschler et al., 2010). In the semilunar valves of mice and chickens, cell density and the proliferative capacity of endocardial cushion cells decreases during leaflet elongation (Kruithof et al., 2007). In mature human leaflets, valvular interstitial cells (VICs) are generally deemed to be quiescent and exhibit a heterogeneous fibroblast-like phenotype (Aikawa et al., 2006). The complex phenotype of VICs implies that they have yet to be fully characterized in detail; however, some general mesenchymal markers including the hyaluronic acid (HA) receptor CD44 are expressed on fibroblast-like VICs (Carthy et al., 2012; Halfon et al., 2011). This receptor is involved in the mediation of cell motility and plays an important role in epithelial-to-mesenchymal transformation (EMT) during cancer progression, which is known to share mechanisms with EndMT during development, and CD44^+^ cells could play a role in leaflet elongation (Kim et al., 2008; von Gise and Pu, 2012; Zoller, 2011).

The role of EndMT in leaflet development has been extensively studied in animal models such as mouse and chicken, and numerous factors involved in its regulation have been identified, including ECM proteins (Norris et al., 2009; Runyan and Markwald, 1983), growth factors (Macgrogan et al., 2011) and various transcription factors. One such transcription factor is nuclear factor of activated T-cells, cytoplasmic, calcineurin-dependent 1 (NFATC1; also known as NFATc-1 and NF-ATC) (Chang et al., 2004; de la Pompa et al., 1998; Ranger et al., 1998). NFATC1 is a calcium-activated transcription factor, which is reported to play an essential role in mouse semilunar valve development (de la Pompa et al., 1998; Ranger et al., 1998). Activated NFATC1 translocates to the nucleus and is predominately expressed in endocardial cells close to the endocardial cushions (Armstrong and Bischoff, 2004; Chang et al., 2004). Furthermore, it has been demonstrated that vascular endothelial growth factor (VEGF)-mediated, calcineurin-activated NFATC1 regulates endothelial cell fate and maintains a valvular endothelial cell (VEC) phenotype (Johnson et al., 2003). Moreover, previous reports postulated that the spatiotemporally distinct function of NFATC1 signaling in SHF cells and endocardial cells is necessary for semilunar cushion formation as well as leaflet elongation and maturation in rodents (Chang et al., 2004; Lin et al., 2012; Wu et al., 2011).

In this study, we performed global gene expression analyses on leaflets from first and second trimester human hearts, which revealed significant differences in the expression profiles of proliferation-associated genes and those associated with EndMT. We therefore hypothesized that human semilunar valvulogenesis is a process that actively begins within the first weeks of development and follows a spatiotemporally defined pattern, in which proliferation decreases during development. We also hypothesized that the replenishment of cells in the mesenchyme is contributed by VECs undergoing EndMT in order to migrate into the cardiac cushions. In addition, we identified expression of CD44 in second trimester valves that begins at the annulus/leaflet junction and progresses towards the leaflet tip during elongation.

## RESULTS

### Cell density and proliferation in developing human semilunar valves decreases from the first to the second trimester

To identify trends in gene expression that change between the first (9-12 weeks) and second (14-17 weeks) trimesters, we employed gene set enrichment analysis (GSEA) on data previously generated by our laboratory (Votteler et al., 2013a). The GSEA revealed a significant number of proliferation-associated gene sets and motifs as enriched in the first trimester compared with the second trimester (Fig. S1, Tables S1 and S2).

**Fig. 1.**
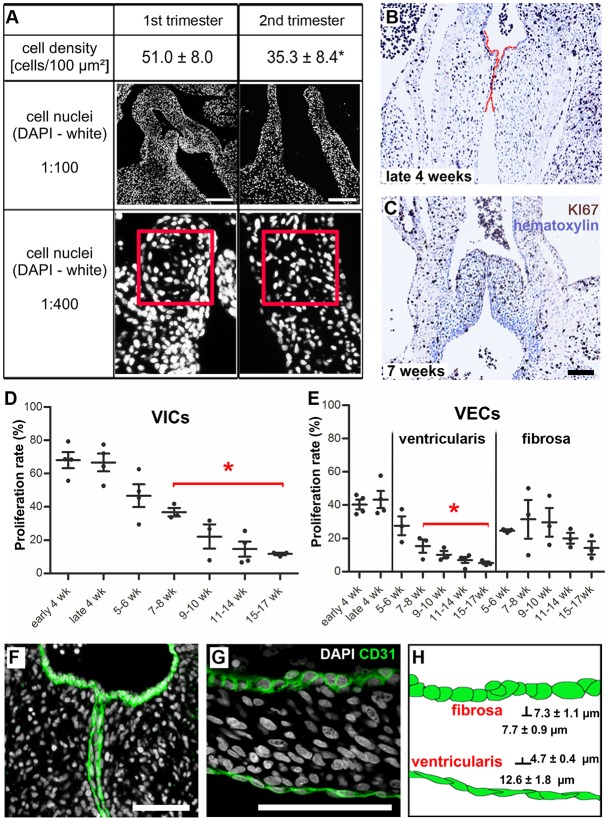
**Cell density and proliferation decrease significantly from the first to the second trimester of leaflet development and defined VEC morphologies are visible as early as 4 weeks.** (A) DAPI staining of human developing cardiac valves shows the different cell densities of the leaflets. The red square is 0.01 mm^2^. *P<0.001. (B,C) Proliferating KI67^+^ VICs (brown) are randomly distributed throughout the fetal cushions and leaflets during developmental stages. (B) Fetal semilunar valve cushions at late 4 weeks, and (C) leaflets at 7 weeks of development. Red lines highlight the semilunar cushions. (D,E) Proliferation rate of VICs and VECs on the ventricularis is significantly decreased beginning at 7-8 weeks of development when compared with late 4 weeks. *P<0.05. (F,G) CD31^+^ VECs in developing valves (green) show characteristically distinct morphologies: VECs with a cuboidal morphology line the fibrosa layer, whereas VECs facing the ventricles are elongated and flattened. (H) Schematic of cell morphologies. VECs of the fibrosa are significantly shorter than VECs of the ventricularis. VEC, ventricular valve endothelial cell; VIC, valvular interstitial cell. Scale bars: 200 µm in A; 100 µm in C,F,G.

To confirm the observations of the GSEA, valve leaflets of first (4-12 weeks, *n*=8) and second (13-17 weeks, *n*=7) trimester hearts were utilized for immunohistological staining. The temporal valvular cell density was quantified by counting cell nuclei in DAPI-stained tissue sections. Similar to patterns in mouse and human tissues of later developmental stages (second and third trimesters) (Aikawa et al., 2006; Hinton et al., 2006; Kruithof et al., 2007), we identified a decrease in cell density during early valve development (Fig. 1A). First trimester cushions and leaflets exhibited 51.0±8.0 cells per 0.01 mm^2^, which was significantly reduced to 35.3±8.4 cells per 0.01 mm^2^ in second trimester leaflets (*P*<0.001). Within these leaflets, the number of KI67^+^ VICs and VECs on the ventricularis was significantly reduced beginning at 7-8 weeks, when compared with late 4 weeks of development (Fig. 1B-E). No discrete spatial patterns of proliferative VICs were evident, as KI67^+^ VICs were randomly distributed throughout the cardiac cushions (Fig. 1B) and elongated leaflets (Fig. 1C). However, it should be noted that there were significantly fewer KI67^+^ VECs on the ventricularis when compared with the fibrosa, a trend that persisted from 7-8 weeks of development (Fig. 1E).

To identify potential mechanisms driving increased proliferation in the first trimester, we screened the GSEA data for likely candidates. Interestingly, several gene sets related to MYC activity were significantly enriched in the first trimester (Fig. S2). As MYC is a known regulator of cell cycle progression (Dang, 1999), we hypothesized that MYC participates in proliferation by upregulating mitosis genes in first trimester leaflets. Comparison of the genes contained within the ‘reactome mitotic M-M/G1 phases' gene set, which was significantly enriched in first trimester leaflets, with a gene set that defined direct MYC targets using ChIP-Seq (Zeller et al., 2003) demonstrated a statistically significant overlap, suggesting that MYC activity influences this enhanced proliferation. Immunohistological staining for MYC protein on first and second trimester leaflets revealed higher levels of MYC in the first trimester, corroborating the gene expression data. Together, these data suggest that increased proliferation in first trimester leaflets is likely to be linked to MYC participation.

### Morphological differentiation of VECs in fetal semilunar valves

Based on the differences in temporal proliferation detected between VECs lining the fibrosa versus the ventricularis, we analyzed these cells in more detail. Previous reports focusing on human postnatal valves demonstrated that VECs lining the ventricularis possess a different morphology to VECs populating the fibrosa layer of the same leaflet (Armstrong and Bischoff, 2004). We established that such VEC morphological differences could be detected as early as week 4 of development (Fig. 1F). We further identified that these cell morphological features were maintained during leaflet maturation. Immunohistological staining revealed a characteristic cuboidal morphology of the CD31^+^ VECs on the fibrosa layer, as opposed to the typical elongated and flattened morphology of the VECs facing the ventricles at 4 and 7 weeks of development (Fig. 1F,G). VECs of the fibrosa exhibited significantly shorter distances between junctions (length) when compared with VECs of the ventricularis (cell length: 7.7±0.9 µm versus 12.6±1.8 µm; *P*<0.001). Correspondingly, VECs of the fibrosa demonstrated a significantly greater basolateral-apical distance (height) when compared with VECs of the ventricularis (cell height: 7.3±1.1 µm versus 4.7±0.4 µm; *P*<0.001) during the first trimester of development (Fig. 1H).

### Gene and protein analyses reveal spatial and temporal changes in NFATC1 expression

**Fig. 2.**
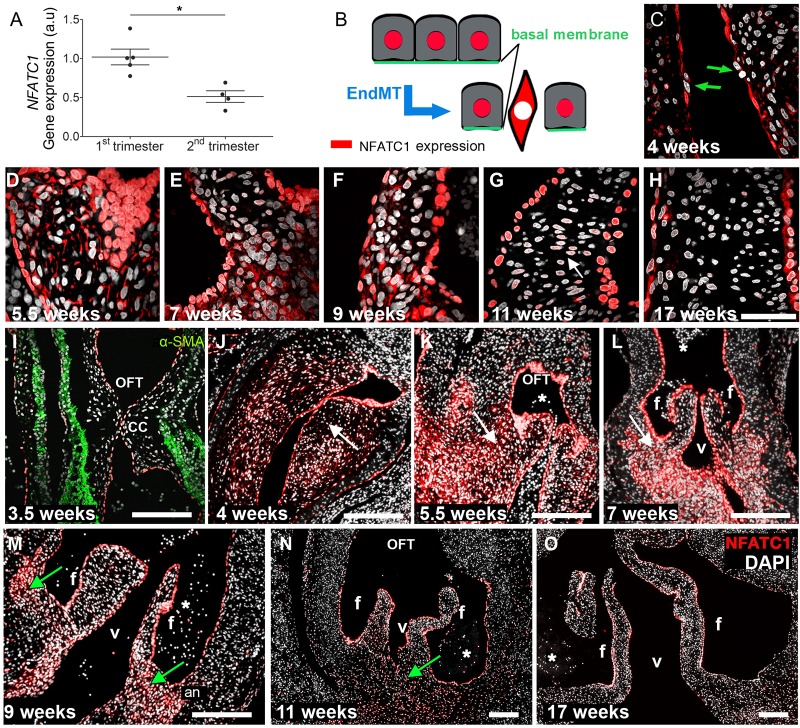
**The spatiotemporal patterns of NFATC1 gene and protein expression.** (A) Gene expression of *NFATC1* in first and second trimester semilunar valve leaflets (*P<0.006, *n*=4). (B) Schematic of spatial NFATC1 expression during EndMT. Active NFATC1 (red), maintaining the endothelial phenotype, is located in the cell nucleus. Although inactive, NFATC1 is expressed in the cytosol. (C-O) Immunofluorescence analyses of NFATC1 protein (red) expression patterns at the indicated stages. DAPI is in white. (C) The green arrows indicate cells appearing to undergo EndMT. (G) White arrow points to cells in the mesenchyme, where NFATC1 expression intensities in the nucleus are significantly decreased compared with endocardial cells. (I-O) During a specific time window between weeks 4 and 8, NFATC1 is also present in the cytosol (white arrows) of cushion mesenchymal cells (J-L). Between week 9 (M) and 11 (N), NFATC1 is expressed in the nucleus (green arrows) of mesenchymal cells at the leaflet annulus. In second trimester leaflets (O), nuclear NFATC1 is predominately found in VECs. an, leaflet annulus; OFT, outflow tract; f, fibrosa; v, ventricularis; CC, cardiac cushions; asterisks, erythrocytes. Scale bars: 50 µm in C-H; 200 µm in I-O.

It is established that the transdifferentiation of VECs through EndMT plays a substantial role in populating the developing cardiac cushion and subsequently elongating the valve leaflet (de la Pompa et al., 1998; Ranger et al., 1998). We observed that *NFATC1* mRNA expression significantly decreased (relative expression: 1.006±0.226 versus 0.513±0; *P*<0.006) from the first to the second trimester of human fetal development (Fig. 2A). Concurrently, we observed changes in NFATC1 protein expression patterns. Once activated, NFATC1 translocates to the nucleus, and cells change their polarity and morphology (Fig. 2B). As NFATC1 is a known repressor of EndMT (Zhou et al., 2005), cells with cytoplasmic NFATC1 could be candidates for VECs changing phenotype to populate the cushions. Notably, we identified individual cells in the endocardium of cardiac cushions at week 4 of development, with cytoplasmic NFATC1 expression, that did not feature the typical morphology of endocardial cells (Fig. 2C, arrows). These data establish that EndMT occurs as early as week 4 in human developing cardiac valve leaflets.

Comparable to reports in mice (de la Pompa et al., 1998; Ranger et al., 1998; Wu et al., 2011), NFATC1 was highly expressed in the nuclei of human endocardial cushion cells at 4-6 weeks of development (Fig. 2C,D), as well as in the nuclei of VECs of elongated leaflets starting as early as 7 weeks of development (Fig. 2E-G). We also detected spatial changes in NFATC1 expression within the leaflet mesenchyme. During cushion formation and the early elongation period (weeks 4-7), we identified strong expression of NFATC1 within the cytoplasm of VICs (Fig. 2D-F). Notably, during elongation, strong cytoplasmic NFATC1 expression was detected in VICs of the leaflet annulus (Fig. 2K,L, arrows). Later in development, between week 9 and early in the second trimester, NFATC1 was also detected in the nucleus of mesenchymal cells, particularly at the leaflet annulus (Fig. 2M,N, arrows). However, the expression intensities [gray value intensity (GVI)] were significantly lower compared with NFATC1 expression in endocardial cells (week 9, endocardium GVI=161±4.1 versus mesenchyme GVI=111.5±5.3; week 11, endocardium GVI=139.6±3.1 versus mesenchyme GVI=60.5±6.2; *P*<0.0001). In accordance with reports that demonstrate NFATC1 regulates endocardial and endothelial cell proliferation (Johnson et al., 2003; Wu et al., 2011; Zhou et al., 2005), we also detected strong nuclear NFATC1 expression in VECs of the elongated leaflets (Fig. 2N,O). These findings are strengthened by the reduced *NFATC1* gene expression seen in the second trimester (Fig. 2A), as cells within the mesenchyme begin to lose NFATC1 expression (nuclear or cytoplasmic) (Fig. 2G,H,N,O).

**Fig. 3.**
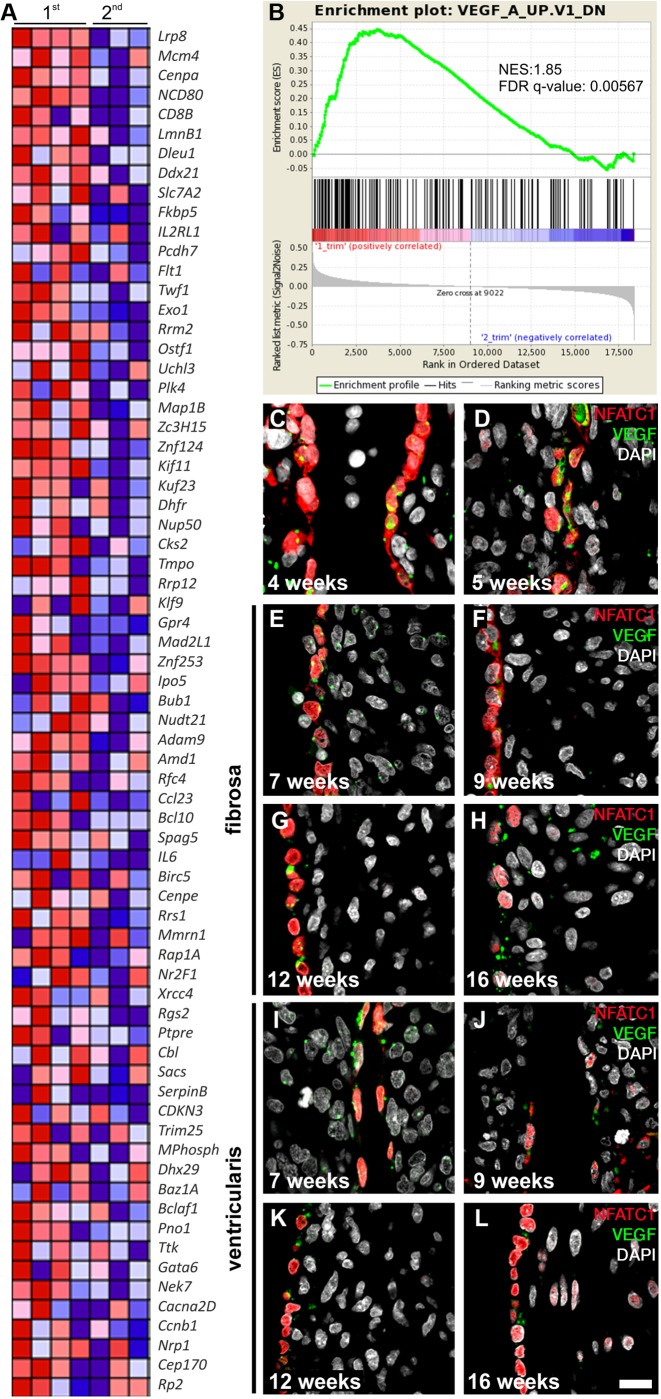
**Increased expression of VEGF targets in the first trimester.** (A) Heat map representing the relative expression and fold enrichment of genes repressed by VEGF (VEGFA) in the first (*n*=4) and second (*n*=3) trimester in OFT leaflets (created using Molecular Signatures Database v5.0). See B for color key. (B) GSEA of enrichment of VEGF repression from leaflets obtained from the first compared with the second trimester. The false discovery rate (FDR) q-value and normalized enrichment score (NES) are shown. (C-L) Immunofluorescence imaging of NFATC1 (red) and VEGF (green) protein expression patterns in early endocardial cushions and elongated leaflets. DAPI is in white. At the early cushion stage (C,D), fibrosa and ventricularis are not yet identifiable. E-H, the fibrosa layer; I-L, the ventricularis. Scale bar: 50 µm.

GSEA revealed that genes repressed by VEGF (VEGFA) signaling are enriched in first trimester valves compared with second trimester valves (Fig. 3A,B). In mice, VEGF has been shown to have a repressive role in regulating EndMT (Dor et al., 2001). As our gene expression data were generated from late first trimester specimens, when EndMT has begun to diminish, this would suggest that the function of VEGF in acting as a brake on EndMT is conserved. We then sought to map the expression of VEGF at the protein level in human semilunar valve leaflet development (Fig. 3C-L). We detected strong VEGF expression in the cardiac cushion VECs (weeks 4-5 of development; Fig. 3C,D), which persisted throughout the course of development. However, no statistically significant trends in protein expression were detected (Fig. S3). Weak VEGF expression was detected within the cushion and leaflet mesenchyme at all time points investigated.

### CD44 expression is clustered at the leaflet/annulus junction and progresses towards the leaflet mesenchyme in second trimester human cardiac valves

**Fig. 4.**
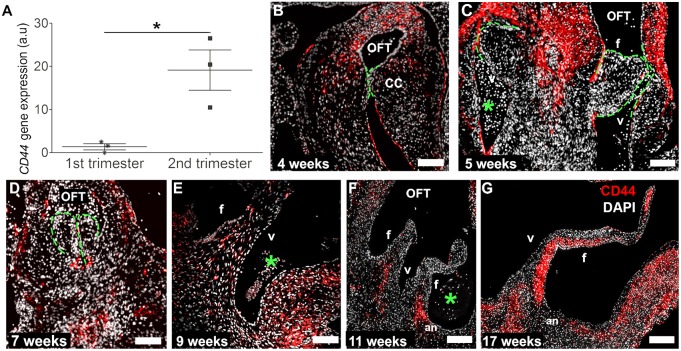
**Increased expression of *CD44* from the first to the second trimester and the spatiotemporal pattern of CD44 protein expression.** *CD44* gene (A; *P<0.05, *n*=3) and protein (B-G) expression analyses reveal the distinct temporal and spatial patterns of CD44^+^ cells (red) during human semilunar valvulogenesis. DAPI is in white. The green lines highlight the semilunar cushions. OFT, outflow tract; f, fibrosa; v, ventricular side; CC, cardiac cushions; an, leaflet annulus; asterisks, erythrocytes. Scale bars: 200 µm in B-E; 400 µm in F,G.

Based on our data showing a significant decrease in VIC proliferation starting at week 7 (Fig. 1D), inactive NFATC1 expression in endocardial cushion cells early in the first trimester (Fig. 2C), and the strong presence of inactive NFATC1 at the annulus prior to elongation (Fig. 2L,M), we sought to determine the contribution of CD44^+^ cells populating the valve leaflets during leaflet development. Quantitative real-time PCR (qRT-PCR) analyses revealed a significant increase (relative expression: 1.374±1.262 versus 19.14±8.081; *P*<0.0003) in *CD44* mRNA expression between first and second trimester leaflets (Fig. 4A). This gene expression pattern was confirmed on the protein level by immunofluorescence staining. CD44 was exclusively expressed on some VECs along the cardiac cushions in 4- to 7-week-old hearts (Fig. 4B-D, Fig. S4). These endothelial cells appear to be assuming a mesenchymal phenotype in order to populate the mesenchyme of the developing valve leaflet. By contrast, CD44^+^ cells were only detectable at 4 weeks of development in the cardiac cushion mesenchyme, and were afterwards present in the myocardial wall (Fig. 4B-D).

**Fig. 5.**
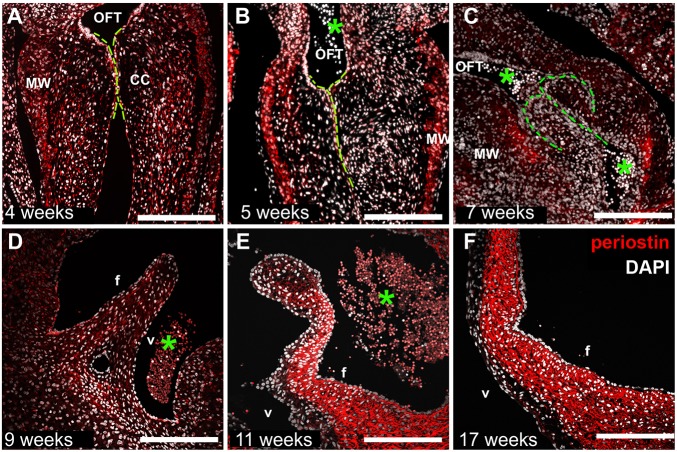
**Periostin expression accumulates strongly in the fibrosal layer of the developing leaflet in the second trimester.** Temporal and spatial distribution of periostin (red) in human first (A-E) and second (F) trimester semilunar leaflets. DAPI is in white. The green lines highlight the semilunar cushions. OFT, outflow tract; asterisks, erythrocytes; f, fibrosa; v, ventricularis; CC, cardiac cushions; MW, myocardial wall. Scale bars: 200 µm in A-D; 400 µm in E,F.

Later in development, we detected spatially distinct expression of CD44 in the developing semilunar leaflets. CD44 expression clustered at the junction between the valve leaflet and annulus (9 weeks, Fig. 4E). Subsequently, at 11 and 16 weeks of development, CD44^+^ cells were detected along the leaflet in a defined domain that progressed toward the leaflet tip over time (Fig. 4F,G). Notably, these CD44^+^ cells were juxtaposed along the fibrosal and spongiosal layer of the elongated leaflets, whereas fewer CD44^+^ cells were detected in the ventricularis. Based on this specific spatial localization, which occurred at defined time points of leaflet development, we hypothesize that signaling through CD44 might contribute to the positioning of these cells. Mesenchymal cells expressing CD44 in other systems are known to migrate through engagement of hyaluronan receptors, and recently an induction of intracellular crosstalk between periostin and hyaluronan has been established (Ghatak et al., 2014), wherein valvular cushion cells were shown to secrete periostin into the ECM *in vitro*, which enhanced hyaluronan expression upon periostin/integrin/focal adhesion kinase-mediated activation of P13K and/or ERK (Norris et al., 2008; Snider et al., 2008). Although the role of periostin in fibrogenesis has yet to be fully clarified, it is agreed that this ECM protein is necessary for the initiation and regulation of collagen deposition. Periostin is detected in mice after EndMT during the development of the atrioventricular (AV) valves (Norris et al., 2008). Here, we report similar findings, with a significant increase in the periostin content after the EndMT that precedes CD44 expression (Ghatak et al., 2014). This expression of periostin becomes extremely marked at 11 to 17 weeks of development compared with earlier time points (Fig. 5A-F), which is when the expression of NFATC1 in the mesenchyme is reduced (Fig. 2G,H,N,O) and the presence of CD44^+^ cells becomes elevated. This time-dependent linkage of CD44^+^ cells with the secretion of periostin suggests that the elongation of the leaflet could be directed by both biochemical and biomechanical cues by either migratory or residential cells.

## DISCUSSION

Our data provide unique insight into the events that support human developmental valvulogenesis. Within this study, we have investigated cellular and molecular processes responsible for human valve maturation and elongation during development. We have identified that human leaflet cell density and proliferation decrease significantly from the first to the second trimester. Differential VEC proliferation patterns were identified in the ventricularis and fibrosa layers. We sought to determine the origin of cells that populate the leaflet mesenchyme during development. We found that VECs undergo EndMT in the cardiac cushions as early as 4 weeks of development, based on inactive cytoplasmic NFATC1 and CD44 expression. Once in the cushions, these cells maintain cytoplasmic NFATC1 expression. Between 5 and 9 weeks of development, we detected strong expression of inactive NFATC1 at the junction of the leaflet/annulus mesenchyme. Later in development (weeks 11 to 17) this expression pattern disappeared and active NFATC1 was only detectable in the VECs. However, during this period (weeks 11 to 17), we saw increased expression of CD44, which clustered at this leaflet/annulus mesenchyme and later appeared along the fibrosal layer of the elongating leaflet. We also identified that this was possibly linked to periostin expression in the second trimester of human valvulogenesis.

Previous studies report that cell density and proliferation are higher in human fetal second and third trimester leaflets compared with mature leaflets (Aikawa et al., 2006). In this present work, we observed a significantly decreasing cell density from the first to the second trimester in human semilunar cushions and leaflets, which is in accordance with findings from studies of valvulogenesis in mice and chickens (Hinton et al., 2006; Kruithof et al., 2007). However, although proliferating cells were detected randomly throughout fetal cushions and leaflets, a specific and significant decrease in the proliferation of ventricular VECs occurred at week 7 of development, which was not detected in fibrosal VECs (Fig. 1E). That there is a difference between VECs of the fibrosa and the ventricularis at 7 to 8 weeks of development is particularly significant. In the human heart, beating begins at ∼4 weeks and accelerates towards a peak of 180 bpm at 7 weeks (Riem Vis et al., 2011). We have previously reported that tropoelastin/elastin deposition is first detectable in the ventricularis of human cardiac valves at this fundamental 7 week milestone (Votteler et al., 2013a). It is therefore interesting to speculate whether VECs of the ventricularis have a significantly decreased proliferation at 7 weeks that could be attributed to hemodynamic differences between the ventricularis and fibrosa.

NFATC1 has been identified as indispensable in rodent semilunar valve development (de la Pompa et al., 1998; Ranger et al., 1998). In this present study, its spatiotemporal expression pattern was analyzed in first and second trimester human cardiac valves in order to establish the contribution of VECs, undergoing EndMT, to leaflet elongation. Nuclear NFATC1 expression was detected in all endocardial cells, with the distinct exception of some single VECs in the cardiac cushions (Fig. 2C, arrows). These particular cells exhibited an altered cell morphology accompanied by NFATC1 being expressed exclusively in the cytoplasm.

In light of the evidence in this study, and that of previous reports (de la Pompa et al., 1998; Johnson et al., 2003; Lin et al., 2012; Ranger et al., 1998; Wu et al., 2011; Zhou et al., 2005), it can be postulated that inactivation of NFATC1 is indicative of EndMT in human endocardial cushion cells. While nuclear NFATC1 was highly expressed in the endocardial cells of early cardiac cushions, and also in VECs of elongated leaflets during all developmental stages, this study also identified specific developmental stages when NFATC1 was detectable in cushion and leaflet mesenchymal cells. During semilunar cushion formation and early elongation, inactive cytoplasmic NFATC1 was observed in mesenchymal cells, particularly at the annulus of the leaflet (Fig. 2K,L). Later during leaflet elongation between week 9 and 11 of development, mesenchymal cells showed activated nuclear NFATC1 in the annulus of the leaflets (Fig. 2M,N). This indicates that, within mesenchymal cells of the developing leaflet annulus, NFATC1 is activated during a specific time frame to quell EndMT. This concurs with the study of Lin et al. (2012), who reported that calcineurin-activated NFATC1 signaling acts in spatiotemporal waves in various tissues during murine semilunar valve development. The same study demonstrated that the role of calcineurin/NFATC1 signaling in the SHF of E7.5 and E8.5 mouse embryos is distinct from its role in the endocardium beginning at E10.5. Whereas calcineurin/NFATC1 signaling in the SHF is required for early semilunar cushion formation by preventing the regression of the cushion mesenchyme (Lin et al., 2012), NFATC1 in the endocardium is required for cushion reorganization and leaflet elongation (Wu et al., 2011). In endocardial cells and VECs, nuclear NFATC1 is required for the maintenance of the VEC phenotype and enhances their proliferation (Johnson et al., 2003), which consequently results in reduced EndMT. Comparable to results from mouse studies (Wu et al., 2011), this mechanism might facilitate the contribution of mesenchymal cells from the leaflet annulus to leaflet elongation. As *Nfatc1* knockout mice fail to develop elongated leaflets (Lin et al., 2012; Wu et al., 2011), we hypothesize that activated NFATC1, which we detected in this study in the cushion mesenchymal cells during weeks 9 and 11 of human cardiac valve development, supports leaflet elongation. This strongly suggests that in humans, NFATC1 is pivotal for semilunar valve development, with distinct roles in endocardial and mesenchymal tissues.

As previously mentioned, it has been demonstrated that VEGF-mediated calcineurin-activated NFATC1 regulates endothelial cell fate and contributes to maintenance of the VEC phenotype (Johnson et al., 2003). However, regulation of leaflet development by VEGF signaling is far from a simple process (Lambrechts and Carmeliet, 2004). VEGF is necessary for initial EndMT; however, it subsequently terminates this process. Initiation and termination of EndMT are both deemed to be VEGF dose dependent and controlled within narrow spatial and temporal windows (Lambrechts and Carmeliet, 2004). In mice, VEGF is detectable in the myocardium and outside the AV canal at E9, which is the time frame at which EndMT begins in mice (Dor et al., 2001). Indeed, it has been shown that lowering VEGF levels at E9.5 via hyperglycemic induction or with a soluble Flt1 chimeric protein prevents EndMT (Enciso et al., 2003). It has also been shown in mouse embryonic explants that EndMT is inhibited by VEGF, through VEGF supplementation and hypoxia-induced VEGF upregulation (Dor et al., 2003). In mice, myocardial VEGF levels in the AV canal are elevated 5- to 10-fold at E10.5 (Dor et al., 2003). These previous studies have established that some VEGF expression is required for endocardial cells to undergo EndMT, but that as EndMT reaches completion, higher levels of VEGF are encountered that halt EndMT. In this study, we observed that *VEGF* gene expression is significantly upregulated in the second trimester of human cardiac valve development, which is in accordance with studies performed in other vertebrates that postulated that high VEGF expression is necessary to terminate EndMT (Dor et al., 2001). Moreover, our findings fit within the time frame of EndMT reduction and termination. VEGF protein expression was evident in the endocardial cushions at all times of development examined; however, we did not detect any statistically significant differences in the expression patterns (Fig. S3).

One of the most significant observations in this study is the contribution of CD44^+^ cells to valve elongation and colonization (Halfon et al., 2011; Hanna et al., 2007). We identified CD44^+^ cells clustering at the annulus of the leaflet during leaflet elongation (Fig. 4F,G), where we had detected a strong expression of inactive NFATC1 (Fig. 2K-M). With ongoing elongation, the presence of CD44^+^ cells extended towards the middle of the leaflet. A number of hypotheses can be put forward to explain the origin of this CD44 expression. CD44 is known to mediate cell motility by HA and epidermal growth factor receptor (EGFR) interaction (Kim et al., 2008). It has been proposed that the condensation of mesenchymal cells in mouse AV valves begins at E15.5, equivalent to week 12 of human development, and expands throughout the leaflet at E18.5, which is approximately equivalent to the third human trimester (Kruithof et al., 2007). Here, in human tissue, a mesenchymal condensation of CD44^+^ cells was first detected at week 11 (Fig. 4F), and subsequently was predominately present in the spongiosa and fibrosa layers. One could speculate that these CD44^+^ cells originate from the previously NFATC1-expressing cells at the same leaflet/annulus junction, which have achieved a more mature mesenchymal cell phenotype, and which then migrate towards the valve tip in a temporal-spatial manner as the leaflet elongates in response to biochemical and biomechanical cues. Indeed, it is also possible that resident VICs, already present in the developing leaflet, begin to express CD44 in response to biophysical stimuli. We have already demonstrated that the VECs of the fibrosa and ventricularis display very different characteristics with regard to cell morphology and proliferation. The same could possibly be true for VICs neighboring these distinct locations. Previously, it has been shown *in vitro* that porcine fibrosal VICs exhibit much lower expression of alpha smooth muscle actin (αSMA) than ventricular VICs that were exposed to the same conditions of cyclic strain (Moraes et al., 2013). A third hypothesis could be put forward that the biomechanical forces in the developing heart elicit biochemical cues from the layer-specific VICs, which facilitate the migration of CD44^+^ cells. Post-EndMT, we identified high expression levels of periostin within the developing human valves. In postnatal valves, expression of periostin is reported to be decreased and mostly present at the ventricular subendothelium (Hakuno et al., 2010). Our investigation of late first trimester and second trimester human tissues does not concur with this postnatal pattern. Here, we detected from 11 weeks of development and persisting in the second trimester at 17 weeks, high and local expression of periostin at the fibrosal layer of the leaflets. The spatial pattern of this periostin expression (Fig. 5) precedes the appearance of CD44^+^ cells (Fig. 4). This suggests that fibrillar ECM deposition begins with the occurrence of CD44^+^ cells at the annulus, progressing towards the leaflet tip along the fibrosal leaflet side via a possible cue of periostin binding or perhaps secretion of periostin by resident fibrosal VICs, in response to other biophysical and biochemical cues. In agreement with previous reports, this periostin expression might stimulate hyaluronan expression (Ghatak et al., 2014), which could facilitate the migration of CD44^+^ cells. Based on published reports of postnatal tissues, it is possible that this periostin expression will extend and persist at the ventricularis (Hakuno et al., 2010).

As cell density significantly decreases during valve development, leaflet growth is primarily due to ECM synthesis and deposition (Hinton et al., 2006); however, utilizing KI67 staining we showed that cellular proliferation also contributes to the elongation of the leaflets, although this is significantly reduced in the second trimester. Therefore, it seems that all processes combined – the migration of VECs into the mesenchyme due to EndMT, the proliferation of VICs within the fetal leaflets, and the presence of CD44^+^ cells at the leaflet/annulus junction and their migration towards the elongating leaflet tip – contribute to leaflet development. This CD44^+^ population is therefore crucial for elongation and maturation towards a trilaminar semilunar leaflet in humans.

Taken together, our study provides unique insights into human semilunar valve development. Similar to previous studies (Chang et al., 2004) of human valvulogenesis, we find that early cardiac cushion invasion by VECs occurs through EndMT and is dictated partially by NFATC1-mediated endocardial and endothelial cell maintenance during valve elongation. Our findings acknowledge the involvement of NFATC1 in early human semilunar valvulogenesis with regard to cushion formation and elongation, and a contribution of VECs to colonization of the mesenchyme in the first trimester. Leaflet elongation in the second trimester is supported by mesenchymal proliferation and the presence of a newly identified CD44^+^ cell subpopulation. All these processes contribute to normal leaflet maturation and stratification.

Owing to the fact that we utilized non-diseased human tissues in this study, we were limited to descriptive analyses that do not provide functional insights. However, this advanced knowledge of early stage human semilunar valvulogenesis will impact research efforts aiming to elucidate mechanisms of congenital valve disease and bring significant insight to studies performed in other vertebrates and *in vitro* models of human development. The rapidly progressing field of tissue engineering and regenerative medicine can harness this information to create relevant disease models, identify potent beneficial pharmacological interventions and possibly create tissue-engineered constructs. Particularly for pediatric valve surgery, the *in vitro* recapitulation of developmental processes will contribute to the future generation of functional tissue-engineered heart valves, which ideally possess the ability to grow and remodel *in vivo*.

## MATERIALS AND METHODS

### Tissue procurement and processing

This study was performed in accordance with institutional guidelines and was approved by the local research ethics committees (UCLA IRB #05-10-093; University Tübingen IRB #356/2008BO2 and #406/2011B02). Human first trimester (*n*=8; 4-12 weeks of gestation) and second trimester (*n*=7; 13-18 weeks of gestation) hearts were obtained from electively aborted fetuses following informed consent and de-identification. After procurement, all tissues were immediately washed in sterile Dulbecco's phosphate-buffered saline. Tissues were then fixed in either 10% phosphate-buffered formalin and embedded in paraffin or directly used for RNA extraction.

### Gene expression analyses

Laser capture microdissection was employed to isolate pure populations of valve leaflet cells and total RNA was extracted using a special isolation kit for formalin-fixed paraffin-embedded samples, as previously described in detail (Votteler et al., 2013b).

Microarray data previously generated by our laboratory (Votteler et al., 2013a) was evaluated at the level of gene sets to define and quantitate trends in gene expression. Ranked gene lists were created and submitted to the online public repository provided by the BROAD Institute for GSEA (Mootha et al., 2003; Subramanian et al., 2005).

qRT-PCR was performed using the QuantiFast Probe one-step assay (Qiagen, Hs_NFATc1_1_FAM QuantiFast Probe Assay, Hs_CD44_1_FAM QuantiFast Probe Assay). We employed 10 ng of total RNA using the manufacturer's recommended cycling conditions (95°C for 3 min followed by 45 cycles at 95°C for 3 s, 60°C for 30 s).

### Immunohistological analyses, semi-quantification and microscopy

Tissue sections were deparaffinized and slides were processed as previously described (Monaghan et al., 2014). The following antibodies were used for immunofluorescence staining: c-MYC (Abcam ab32072, 1:100), NFATC1 (Santa Cruz sc-7294, 1:1000), CD31 (PECAM1; Santa Cruz sc-71872, 1:1500), VEGF (Thermo Fisher Scientific RB-9031-P, 1:4000) and the Prestige antibody CD44 (Sigma-Aldrich HPA005785, 1:3500). For NFATC1, CD31, VEGF and CD44 detection, we performed amplified immunofluorescence staining using Tyramide Signal Amplification kits (T20911 and T20915, Life Technologies). After the primary antibody detection procedure, slides were exposed to a DAPI solution for 10 min, followed by mounting using ProLong Gold antifade mounting medium (Molecular Probes, Life Technologies). Fluorescence images were acquired using an Axio Observer Z1 (Carl Zeiss) or an LSM 710 confocal microscope (Carl Zeiss). Images were processed with Photoshop CS5 (Adobe Systems).

Immunohistochemical staining of the proliferation marker KI67 (MKI67) (antibody: Leica Biosystems MIB I, KI67-MM1-L-CE, 1:100) was kindly performed by the pathology laboratory of Prof. Dr Burkhard (Reutlingen, Germany). The staining procedure was conducted automatically using the staining machine BOND-MAX according to the manufacturer's suggested protocol (Leica Biosystems).

For semi-quantification of NFATC1 protein expression levels, GVIs were measured by densitometry and analyzed using ImageJ software (NIH) as described (Schesny et al., 2014). All data are displayed as mean±s.d. of results obtained from 20 cells for each cell phenotype in each sample.

### Assessment of cell density

Cell density was calculated as the mean number of cells from a minimum of 20 DAPI-stained heart valve cushion and leaflet sections using high-power magnification (400×). The cell number is reported as cells per 0.01 mm^2^ within the cushion and leaflet tissue section.

### Analysis of statistical significance

Statistical significance was determined by one-way ANOVA followed by Tukey's multiple comparison tests and Student's *t*-test using GraphPad Prism 5 software. *P*<0.05 was defined as statistically significant.
